# Quantitative color profiling of digital images with earth mover’s distance using the R package colordistance

**DOI:** 10.7717/peerj.6398

**Published:** 2019-02-06

**Authors:** Hannah I. Weller, Mark W. Westneat

**Affiliations:** 1 Department of Organismal Biology and Anatomy, University of Chicago, Chicago, IL, USA; 2 Department of Ecology and Evolutionary Biology, Brown University, Providence, RI, USA

**Keywords:** R packages, Color, Image processing, Phylogenetics, Camouflage, Earth mover’s distance, Statistics

## Abstract

Biological color may be adaptive or incidental, seasonal or permanent, species- or population-specific, or modified for breeding, defense or camouflage. Although color is a hugely informative aspect of biology, quantitative color comparisons are notoriously difficult. Color comparison is limited by categorization methods, with available tools requiring either subjective classifications, or expensive equipment, software, and expertise. We present an R package for processing images of organisms (or other objects) in order to quantify color profiles, gather color trait data, and compare color palettes on the basis of color similarity and amount. The package treats image pixels as 3D coordinates in a “color space,” producing a multidimensional color histogram for each image. Pairwise distances between histograms are computed using earth mover’s distance, a technique borrowed from computer vision, that compares histograms using transportation costs. Users choose a color space, parameters for generating color histograms, and a pairwise comparison method to produce a color distance matrix for a set of images. The package is intended as a more rigorous alternative to subjective, manual digital image analyses, not as a replacement for more advanced techniques that rely on detailed spectrophotometry methods unavailable to many users. Here, we outline the basic functions of colordistance, provide guidelines for the available color spaces and quantification methods, and compare this toolkit with other available methods. The tools presented for quantitative color analysis may be applied to a broad range of questions in biology and other disciplines.

## Introduction

Color is an information-rich trait, and has provided countless insights in biology, including into camouflage, mimicry, pollination, signaling, mate attraction, pathogen infection, and thermoregulation (Cuthill et al., 2017; Liu & Nizet, 2009; Clegg & Durbin, 2000; Smith & Goldberg, 2015; Smith et al., 2016; Bechtel, Rivard & Sánchez-Azofeifa, 2002; Lev-Yadun et al., 2004; Pérez-De la Fuente et al., 2012; Stevens, Lown & Wood, 2014; Chiao et al., 2011; Brady et al., 2015; Troscianko et al., 2016). Unlike many other informative traits, collecting color information can be minimally invasive, and can be done with inexpensive, commercially available digital cameras. Although the resulting digital images are intended to mimic human vision, appropriate calibration and an understanding of these limitations can allow scientists to answer a much wider range of questions with this simpler data format (Troscianko & Stevens, 2015).

Despite the questions surrounding the role of coloration in ecological and evolutionary processes, color is notoriously difficult to categorize. Classifications are often subjective, especially when trying to compare organisms with highly variable appearances. Any objective categorization must account for the amount, distribution, classification, and variety of colors consistently across a set of images. Researchers must also account for the limits of using digital images to answer questions about the visual systems of non-human animals. Common approaches to color profiling often address one or several of these problems, and include qualitative categorization (Puebla, Bermingham & Whiteman, 2007), analysis of digital photographs using pixel color spectra (Byers, 2006), binary character matrices scoring color presence (Marshall et al., 2003), and quantitative point spectrophotometry (Badiane et al., 2017; Safran & Mcgraw, 2004; Marshall et al., 2003). Generally, more comprehensive methods require expensive equipment, expertise, and coding skills, while more straightforward methods are tailored for specific studies, giving them a more limited scope.

Recently, software toolboxes have been gaining popularity as accessible, comprehensive, and consistent methods for image analysis (Troscianko & Stevens, 2015; Bradski, 2000), including a number of R packages. R is among the most popular coding languages for biologists, partly because it is user-friendly and open-source. Although there are several R packages designed for digital image analysis (Van Belleghem et al., 2017; Maia et al., 2013; Barthelme, 2017; Carlson, 2016), to our knowledge, none of them provide methods for profiling and quantitatively comparing colors across images in multiple color spaces.

Here, we present a quantitative approach to color profiling and comparison with digital images in an R package, colordistance, which provides a viable, statistically rigorous option for color profiling and comparison in a user-friendly format (R Core Team, 2018). Although the standard red-green-blue (RGB) format of digital images is a poor proxy for non-human vision (Vorobyev et al., 2001; Endler, 2012; Troscianko & Stevens, 2015), appropriate image calibration and color space conversion can still provide meaningful biological insights with a lower barrier to entry than spectrophotometric methods, and can reflect the visual sensitivities of many species (Losey et al., 2003; Marshall et al., 2003).

Colordistance provides an objective comparative tool for any color analysis that might otherwise rely on a more subjective classification scheme. The package also comes with a pipeline function for streamlined analysis. The central aims of this method are (1) to enable the user to quickly quantify colors in images of organisms (or other objects), (2) to provide tools for categorizing diverse color palettes into bins of similar colors and quantify their extent on a surface, and (3) to develop approaches for color profile comparison and assessment of “color distance,” a metric that borrows techniques from computational image processing to measure the difference in color between objects (Zhang, Barhomi & Serre, 2012; Byers, 2006; Phung et al., 2005; Scheunders, 1997). Colordistance is not meant to replace more comprehensive methods of color comparison, but to provide a more objective, consistent, and easy-to-use alternative to manual classifications. It can also be used to supplement other methods that address different aspects of color diversity in organisms.

## Materials and Methods

### Package details

Colordistance includes 29 exported functions, the most central of which are listed in Table 1. Colordistance imports or suggests R packages for image analysis and data clustering, including jpeg (Urbanek, 2014), png (Urbanek, 2013), clue (Hornik, 2005), spatstat (Baddeley, Rubak & Turner, 2015), ape (Paradis, Claude & Strimmer, 2004), mgcv (Wood, 2011), emdist (Urbanek & Rubner, 2012), scatterplot3d (Liggs & Mächler, 2003), plotly (Sievert et al., 2017), gplots (Warnes et al., 2016), and abind (Plate & Heiberger, 2016).

**Table 1:** Primary colordistance functions and descriptions.

Function	Description
loadImage	Import image as 3D array and generate filtered 2D pixel array(s) of non-masked objects
convertColorSpace	Convert pixels between different color spaces (CIE Lab, RGB, and HSV)
plotPixels	Plot pixels from an image in color space
getImageHist and getHistList	Generate a 3D histogram based on color distribution in an image (or list of histograms for a set of images)
getKMeanColors and getKMeansList	Generate color clusters using k-means clustering for an image (or list of clusters for a set of images)
combineList	Combine a list of cluster features into a single cluster set
getColorDistanceMatrix	Generate a distance matrix for a list of color histograms or cluster sets
imageClusterPipeline	Generate and plot a color distance matrix from a set of images

A stable distribution of the colordistance package can be downloaded for free at https://CRAN.R-project.org/package=colordistance, and the development version and installation instructions can be found at https://github.com/hiweller/colordistance, along with a forum for user feedback and suggestions. A series of explanatory vignettes providing more detailed explanations and examples is available at the corresponding GitHub Pages site, https://hiweller.github.io/colordistance/. Questions or issues can be posted on https://github.com/hiweller/colordistance/issues.

The CRAN version of the package can be installed by running the following line of code in the R console:

> install.packages(“colordistance”)    1

The main work flow of colordistance consists of three steps:

1. *Image preparation.* Quality color images (JPEG or PNG) of the object(s) of interest are obtained, color calibrated, and backgrounds are masked out with a uniform color, using an image editor outside of the R environment. See below for a discussion of image calibration.
2. *Color binning.* Images are read into R as 3D arrays, and non-background pixels are binned into color categories via one of two provided binning methods to produce a normalized color space histogram.
3. *Histogram comparisons.* Earth mover’s distance (EMD) (Rubner & Tomasi, 2013) or another metric is used for pairwise comparisons of histograms from a set of images, resulting in a distance matrix summarizing the color distance score between each pair of images.

The most important user-specifiable parameters for the analysis are provided in Table 2.

**Table 2:** User-specifiable parameters in colordistance analyses.

Parameter	Function	Options
Color space	One of three common three-component color spaces used in digital images	CIE Lab, red-green-blue (RGB) or hue-saturation-value (HSV)
Background color	Color(s) to be ignored in analysis	Any color range specified by the user
Binning method	Method for grouping pixels in organism/object into bins to summarize and compare images	Color histogram or k-means clustering
Bins	How to divide up color space so that pixels assigned to the same bin are grouped into one color	Either a number of bins per color space channel (if using color histogram) or a total number of clusters (if using k-means clustering)
Color distance metric	Method for calculating the distance between one binned image and another	Earth mover’s distance, χ^2^ distance, Euclidean color distance, or a weighted combination

### Image preparation and calibration

Digital cameras are an accessible, affordable, and non-invasive method of data collection. The resulting images, however, are optimized for human vision and for display on commercial RGB monitors. The actual spectral reflectance of the photographed object is therefore distorted in a digital image. Accurate image calibration, including white balance, radiance normalization, and converting to the color sensitivities of non-human animals, is an essential step before image analysis. A comprehensive discussion of image calibration is beyond the scope of this paper, but see Troscianko & Stevens (2015), Byers (2006), Endler & Mielke (2005) and Schindelin et al. (2012).

Because colordistance does not include image calibration tools, images should be calibrated before being analyzed in R. There are a variety of tools available for image calibration, including simple white-balance correction in most image editing applications. The image calibration and analysis ImageJ toolbox by Troscianko & Stevens (2015) allows users to not only calibrate images, but also to correct for the non-linearity of RGB images and to incorporate ultraviolet (UV) channels to simulate animal color vision; the plug-in is free and comes with a comprehensive guide for users with camera RAW images.

Background masking is the last step of image preparation. Any part of an image that the user wants to ignore should be masked out with a uniform background color that is not similar to any of the colors in the object itself; the examples below use bright green (RGB triplet of (0, 1, 0) on a 0–1 scale) and white (RGB triplet of (1, 1, 1)). This can be accomplished with Photoshop, ImageJ, or other image editing software.

### Color spaces, binning methods, and distance metrics

No universal set of parameters will produce optimal results for all datasets. Instead, colordistance provides several options for each step of an analysis (Table 1). The functions come with defaults that act as useful starting points, but understanding how each parameter will affect the outcome is crucial for accurately interpreting results. See Discussion for suggestions on when to use which options.

#### Color space

The three available color spaces in colordistance are CIE Lab (luminance, red-green, and blue-yellow channels), RGB (red, green, and blue channels), and HSV (hue, saturation, and value channels). The advantages and disadvantages of each color space are discussed more thoroughly both in the discussion and in the “Color Spaces” vignette that comes with the package (also accessible on the CRAN repository).

**Figure 1:**
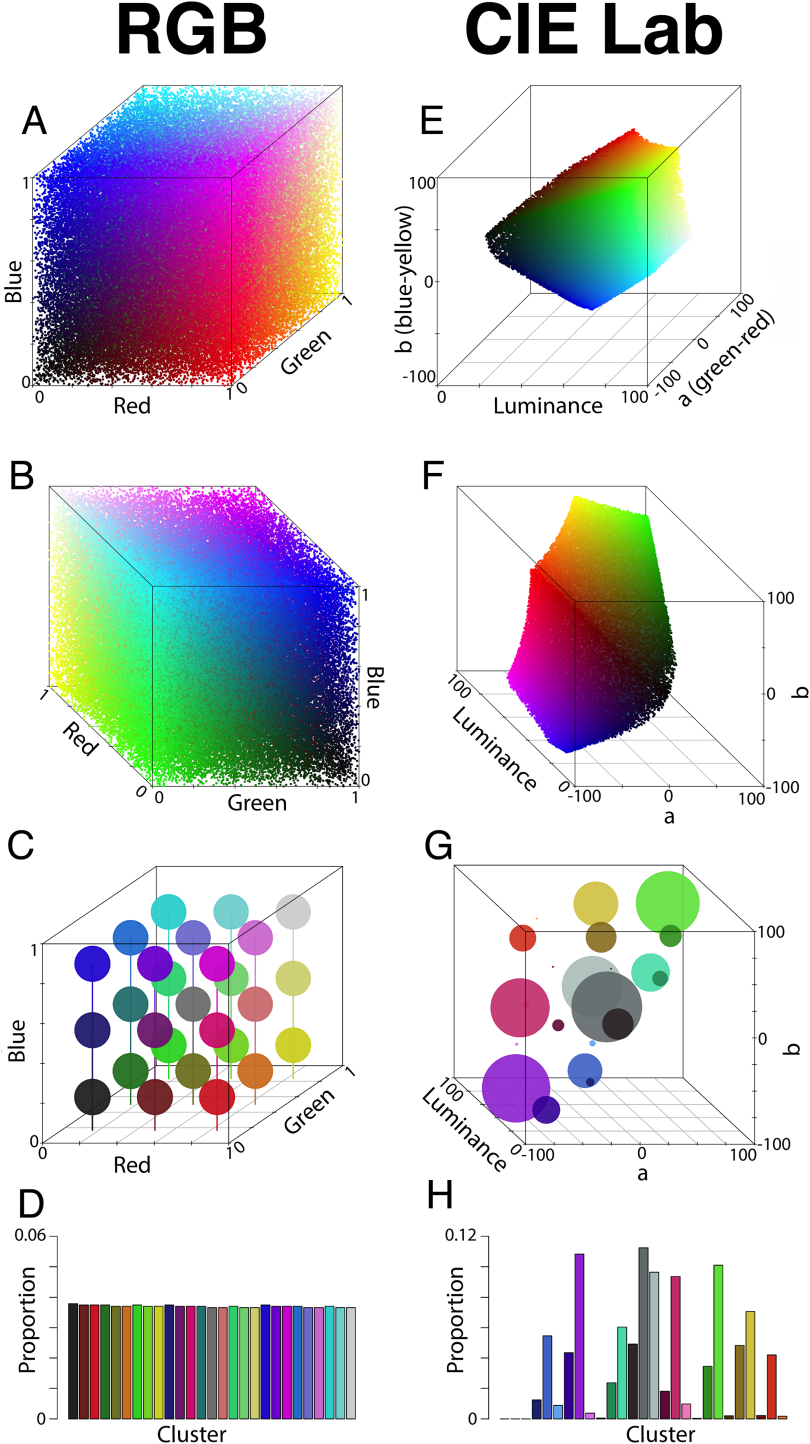
RGB colors as displayed in RGB and CIE Lab color spaces. (A–D) A total of 100,000 random RGB pixels as displayed and clustered in RGB space. (A) and (B) Pixels plotted in RGB space, viewed from different angles; (C) clustering results for binning pixels into 27 equally spaced bins; (D) histogram representation of the clusters in C. (E–H) Same as (A–D) but in CIE Lab rather than RGB space.

Briefly, CIE Lab is a perceptually uniform, device-independent color space, meaning that Euclidean distances between colors in CIE Lab-space reflect the degree of perceived difference between those colors in human color vision. RGB is also modeled on human color vision, but is not perceptually uniform, and is largely optimized for digital displays. HSV color space is intended largely for color manipulation and is not modeled on perception, but is useful for image segmentation for analyses that are not concerned with replicating animal color vision (Hill, Roger & Vorhagen, 1997). Figure 1 illustrates how standard RGB pixels are distributed very differently in RGB and CIE Lab color spaces. In colordistance, RGB color space is set as the default color space, but RGB analyses come with warnings about perceptual non-uniformity to encourage users to read about and implement CIE Lab analyses instead.

#### Binning methods

The two methods for binning pixels, histogram and *k*-means clustering, are fairly common approaches to cluster analysis. Briefly, *k*-means clustering partitions pixels in color space into a specified number of bins in order to minimize the overall sum of pixel-center distances. Though popular, this method can be fairly slow and the cluster locations will be biased toward dominant colors. The histogram method (default) divides a 3D color space into regions depending on user-specified boundaries, computes the proportion of pixels and average pixel value in each region to produce a 3D histogram whose bin centers will vary from image to image. This method is typically faster and not biased by color proportions, but risks breaking up a single color cluster across multiple boundaries.

The *a* and *b* channels of CIE Lab color space are theoretically unbounded, but in practice, RGB colors converted to CIE Lab space have *a* and *b* values between −128 and 127 (Hill, Roger & Vorhagen, 1997); these are used as the upper and lower bounds for each channel unless otherwise specified.

#### Distance metrics

Colordistance includes four color distance metrics, but the most comprehensive is the earth mover’s distance (EMD). The EMD or Wasserstein metric measures the distance between two distributions as a transport cost—essentially, what is the minimum cost of transforming one distribution into the other (Rubner, Tomasi & Guibas, 2000)? It takes into account both spatial color information and size information. For colordistance, when using RGB color space, EMD also has the advantage of having a consistent lower and upper bound. The maximum EMD score in RGB space is $\sqrt 3 $, which is the cost of moving all of the data (*p* = 1) as far as possible across RGB or HSV color space (the diagonal of a cube with sides of length 1). χ^2^ distance also performs well in many cases, but treats bins as independent of each other, so it can result in higher color distances when images have similar colors that are binned differently (i.e., an all-black and all-gray image will have the same distance as an all-black and all-white image). EMD is therefore the default. Other distance metrics are discussed in the “Distance metrics” vignette, which comes with the package or can be found at https://cran.r-project.org/web/packages/colordistance/vignettes/color-metrics.html.

### Implementation

All examples in this paper can be reproduced by cloning the colordistance_examples GitHub repository (http://github.com/hiweller/colordistance_examples) and setting the R working directory to that folder. Lines preceded by “>” indicate commands executed in the R console.

> library(colordistance)            1
 > setwd(“[path/to/directory]/Examples”)   2

**Figure 2:**
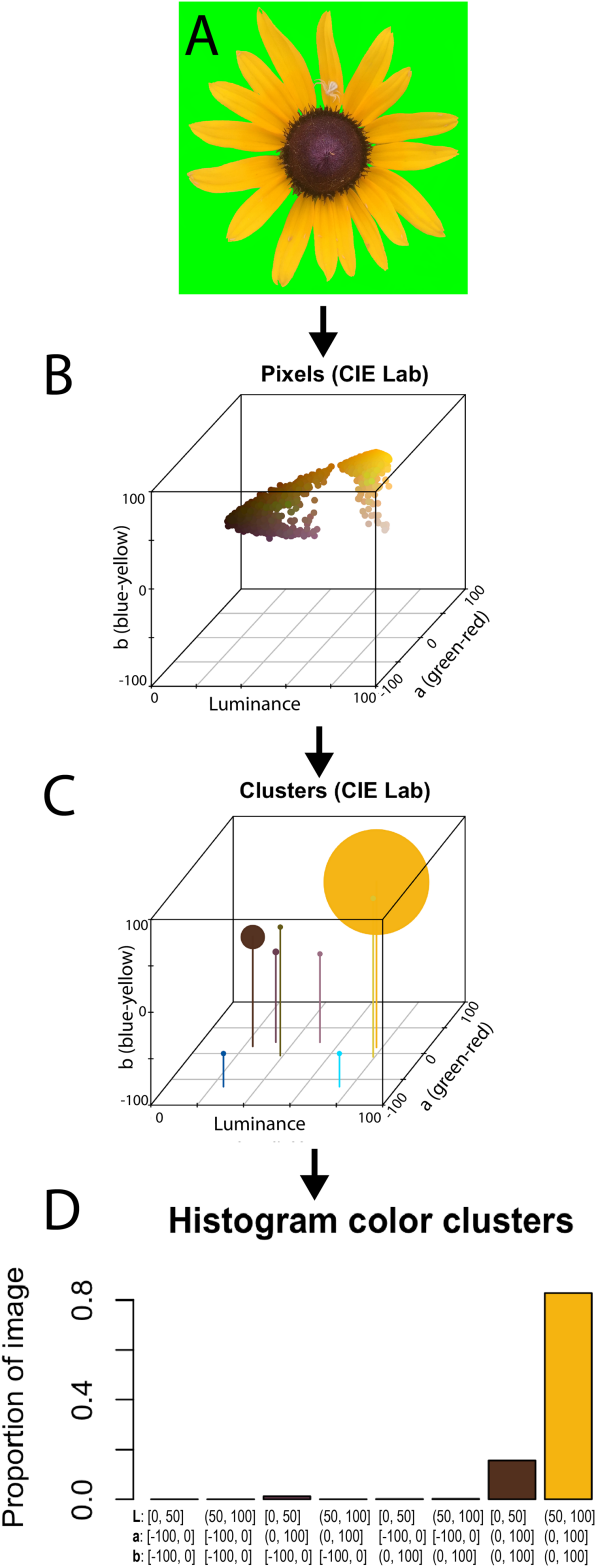
Color binning of a single object. (A) Image of a flower with a background mask of bright green pixels (RGB triplet value (0, 1, 0)); (B) 3D scatterplot of all non-background pixels in CIE Lab color space using plotPixels function; (C) clusters from the histogram in (B) displayed in CIE Lab color space; (D) histogram from getLabHist function showing the proportion of total non-background pixels assigned to each of eight bins, with the color ranges of the bins on the *X* axis. The vertical lines in D indicate the *X* and *Y* (Luminance and a channel) positions of each cluster; the size of each cluster has been increased by 3% so that the locations of empty clusters are still visible. Bins in (C) and (D) have been colored by the average color of the pixels in each bin. Photo credit: H. Weller.

Figure 2 illustrates how the package handles a single image. Prior to loading the image into colordistance, the background of the photograph has been masked out using pure green, which has an RGB triplet of (0, 1, 0) (Fig. 2A). The plotPixels function can be used to visualize the distribution of the flower’s colors in CIE Lab color space. In order to plot the flower in CIE Lab color space (Fig. 2B), we provide plotPixels with: (1) the path to the background-masked image, (2) lower and upper bounds for RGB pixels to ignore, (3) the color space in which to plot, and (4) the name of a standard reference white for RGB to CIE Lab conversion, since the image is stored in an RGB format.

> plotPixels(“Flower/flower_greenscreen.jpg”,            1
     lower = c(0, 0.6, 0), upper = c(0.4, 1, 0.4),       2
     color.space = “lab”, ref.white = “D65”)           3

The lower and upper arguments passed to plotPixels are the lower and upper bounds for background pixels; any pixel with 0 ≤ R ≤ 0.4, 0.6 ≤ G ≤ 1, and 0 ≤ B ≤ 0.4 will be ignored.

The getLabHist function sorts each non-background pixel in the image into a bin, with boundaries defined by the bins argument. Line 1 uses two bins per channel, meaning each of the luminance, *a* (red-green), and *b* (blue-yellow) channels is divided at the halfway point, resulting in 2^3^ = 8 bins. The a.bounds and b.bounds arguments bound the *a* and *b* channels at −100 and 100, rather than −128 and 127. These bounds were chosen because none of the pixels in the image fall outside of these bounds, and narrowing the upper and lower limits reduces the number of empty bins.

> image_histogram <- getLabHist(“Flower/flower_greenscreen.jpg”,    1
   lower = c(0, 0.6, 0), upper = c(0.4, 1, 0.4),      2
    a.bounds = c(-100, 100), b.bounds = c(-100, 100),  3
    bins = c(2, 2, 2), plotting = TRUE, ref.white = “D65”)      4

Binning the pixels produces a three-dimensional histogram, with the location of each bin determined by the average value of the pixels in that bin, and the size determined by the proportion of total pixels in the bin, ranging from 0 to 1. Figure 2C illustrates the relative size and location of each bin in CIE Lab space, while 2D is the diagnostic histogram produced by getLabHist. Each histogram bin represents one of the spheres in 2C.

> print(image_histogram)      1
  L  a  b  Pct          2
1 25.00 -64.25 -64.25 0.00     3
2 75.00 -64.25 -64.25 0.00     4
3 31.46 21.93 -2.78 0.01      5
4 54.81 23.00 -6.52 0.00      6
5 40.14 -6.82 40.77 0.00      7
6 74.86 -26.66 68.94 0.01      8
7 23.72 13.98 18.18 0.15      9
8 77.16 11.82 77.82 0.82      10

The first three columns in the resulting R dataframe represent the average color coordinates of all pixels in a bin; if no pixels were assigned to that bin (as in bins 1, 2, 4, and 5), the center of the bin is used. The last column, percent, represents the proportion of pixels assigned to that bin. For example, the yellow petals of the flower, which fall into bin 8, have a high average luminance (*L* = 77.16 on a 0–100 scale), don’t skew particularly red or green in the *a* (red-green) channel (11.82 on a −100–100 scale), and are much more toward the yellow end of the *b* (blue-yellow) channel (77.82 on a −100–100 scale). They also make up 82% of the image. Histograms are generated for every provided image and a pairwise distance matrix is computed for the image set, providing a quantitative measure of color palette similarities.

### Comparison with patternize

We analyzed the same set of images with colordistance and patternize (Van Belleghem et al., 2017) to illustrate the differences between the two packages. Wherever possible, we chose options in patternize that were comparable to the methods provided by colordistance in order to provide a reasonable basis for comparison. Because the dataset in question (images of five species of parrotfishes) have substantial variety in color, pattern, and body shape, we used landmark alignment rather than Procrustes alignment of patterns to align the images.

Because all of our images were lateral views of the fish, we chose 11 homologous landmarks for alignment in patternize: (1) the center of the eye; (2)–(3) the bases of the first and last fin rays of the dorsal fin; (4)–(6) the bases of the dorsal, midline, and ventral fin rays of the caudal fin where they meet the caudal peduncle; (7)–(10) the bases of the first and last fin rays of the anal and pelvic fins; (11) the anterior tip of the lower jaw. Landmark locations expressed as pixel coordinates for each image were stored as text files, and the backgrounds of the images themselves were masked out with white.

To perform pattern analyses, patternize requires either the specification of an RGB triplet with which to define a pattern or the use of *k*-means clustering to find patterns automatically. *K*-means clustering does not necessarily return a set of colors that are comparable across images, since not all images in the dataset share a color palette, so we chose to manually specify RGB colors. Colors were chosen by selecting patches of a given color in an image and finding the average RGB value, then adjusting the color offset (i.e., allowed deviance from the specified color) until the full color pattern appeared to be captured for each image.

The chosen colors were green-blue (RGB: 0.08, 0.47, 0.43), orange (RGB: 0.78, 0.47, 0.31), pink (RGB: 0.82, 0.59, 0.57), and brown (0.57, 0.49, 0.33). The patLanRGB function was called for the images for each color, and principal component analysis was performed using patternize’s patPCA function. To combine the four sets of PCA results into a single distance matrix for comparison with colordistance, the distance between each pair of images for each color was found by measuring the Euclidean distance between each pair of principal component scores, and the values of the distance matrices were then averaged to produce the final set of similarity scores.

To analyze the same dataset in colordistance, images were analyzed in CIE Lab colorspace with a D65 reference white. The *a*- and *b*-channel ranges were restricted to the range exhibited by the images themselves (see example 2, below), but otherwise, default parameters were used.

## Results

### Benchmarking

#### Earth mover’s distance

**Figure 3:**
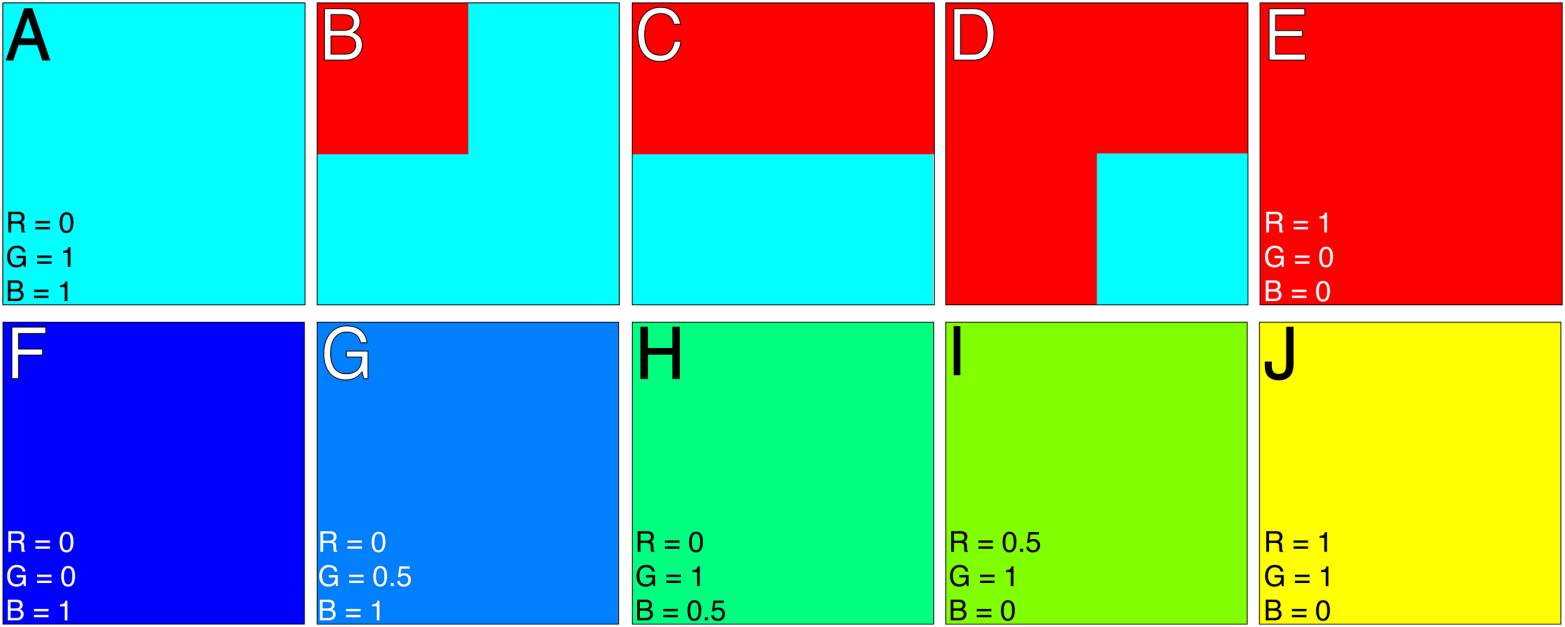
Artificial color images for testing colordistance’s ability to discriminate color quantity (A–E) and color similarity (F–J). RGB triplets are given in the lower left-hand corners. (A–E) Varies the relative amounts of red and cyan in each square: (A) Completely cyan; (B) ${3 \over 4}$ cyan, ${1 \over 4}$ red; (C) ${1 \over 2}$ of each color; (D) ${3 \over 4}$ red, ${1 \over 4}$ cyan; (E) completely red. (F–J) Varies the entire square color on a blue-yellow gradient. Note that for each set, the extremes (A), (E), (F), and (J) are on opposite ends of RGB color space.

We created two simple image sets with known RGB values and proportions (Fig. 3) to test whether the colordistance application of EMD provides scores that accurately reflect the amount and similarities of colors across images. The first set (Figs. 3A–3E) varies the relative proportions of two colors, cyan (RGB triplet of 0, 1, 1) and red (1, 0, 0), and was designed to test whether the distance scores provided by colordistance reflect the differences in the quantities of colors in an image set. The second set (Figs. 3F–3J) samples a gradient from blue (0, 0, 1) to yellow (1, 1, 0), and was designed to test whether scores reflect the relative similarities of colors in an image set. The pipeline function (Table 1 and see below) was used to test each set in both RGB and CIE Lab color spaces:

> imageClusterPipeline(‘Benchmark/Color_quantity’,                   1
          color.space = “rgb”, distance.method = “emd”)            2
> imageClusterPipeline(‘Benchmark/Color_quantity’,                   3
          color.space = “lab”, ref.white = “D65”, distance.method = “emd”) 4
> imageClusterPipeline(‘Benchmark/Color_similarity/’,                  5
          color.space = “rgb”, distance.method = “emd”)            6
> imageClusterPipeline(‘Benchmark/Color_similarity/’,                  7
          color.space = “lab”, ref.white = “D65”, distance.method = “emd”) 8

Distance matrices using EMD were calculated for both RGB space and CIE Lab space. Because RGB space is a cube with sides of length 1, the maximum EMD score should be the length of the diagonal of the cube $(\sqrt 3)$ multiplied by the maximum proportion of pixels that can be separated by this distance (*p* = 1). RGB space was used here, because it has a known maximum score in EMD. CIE Lab space cannot be scaled universally, partly because the maximum score will depend on the conversion parameters, and partly because the shape occupied by visible colors in CIE Lab space is asymmetrical (Figs. 1E and 1F). Scores are typically below 250.

Square color distance matrices are shown in Tables 2 through 5, with distances expressed as proportions of $\sqrt 3 $ for RGB space. The pairs of extremes for each set (A and E; F and J) scored 1, the maximum distance, as expected for colors on opposite ends of RGB color space (Tables 3 and 5). For images A–E, the distance scores between image pairs reflect the proportions of each color in each: Figs. 3A and 3B have a low distance score of 0.25, reflecting the fact that ${1 \over 4}$ of B is red while the rest is the same color as A, as are D and E. Figure 3C is ${1 \over 2}$ of each color, and as expected is half-maximal distance from each of A and B. Although the EMD scores for CIE Lab space (Table 4) are considerably higher, the relative proportions are the same, with the lowest score (40) being approximately ${1 \over 4}$ the maximum score (157), and Figs. 3A and 3E having the highest score.

**Table 3:** RGB pairwise colordistance matrix for Figs. 3A–3E normalized to $\sqrt 3 $, the maximum EMD score for RGB space.

	A	B	C	D	E
A	–	–	–	–	–
B	0.25	–	–	–	–
C	0.50	0.25	–	–	–
D	0.75	0.50	0.25	–	–
E	**1.0**	0.75	0.50	0.25	–

**Note:**

Maximum score is in bold.

Similarly, for the color gradient in Figs. 3F–3J, F and J received the maximum distance score of 1 (Table 4), with images I and J and images F and G receiving lower distance scores of 0.28 in RGB space, reflecting their closer color similarities. Figure 3H scores as equidistant from either F or J with a distance score of 0.64 from either extreme. Unlike in A–E, where C was exactly half-maximal distance from either extreme, the green square in H is not precisely halfway between F and J in color space, and so has a distance score of > 0.5. The computed color distances reflect the known RGB distances of the squares on a quantified scale. Note, however, that for CIE Lab space, the maximum distance score is between Figs. 3F and 3I, rather than F and J. This is because blue and yellow RGB values occupy opposite ends of the *b* channel (blue-yellow) of CIE Lab space, and both have very high luminance values (*L* = 90 and *L* = 97 for blue and yellow, respectively).

**Table 4:** CIE Lab pairwise colordistance matrix for Figs. 3A–3E.

	A	B	C	D	E
A	–	–	–	–	–
B	40	–	–	–	–
C	78	40	–	–	–
D	118	78	40	–	–
E	**157**	118	78	40	–

**Note:**

Not normalized because there is no absolute maximum EMD score in CIE Lab space. Maximum score is in bold.

**Table 5:** RGB pairwise colordistance matrix for Figs. 3F–3J, normalized as in Table 3.

	F	G	H	I	J
F	–	–	–	–	–
G	0.28	–	–	–	–
H	0.64	0.40	–	–	–
I	0.86	0.70	0.40	–	–
J	**1.0**	0.86	0.64	0.28	–

**Note:**

Maximum score is in bold.

For both color spaces, EMD scores reflect differences in both amount and similarity of colors in the images.

#### Function timing

The most time-consuming functions in colordistance are those that directly process or handle images, including loading the images, converting between color spaces, and binning. To time these functions, we generated random square RGB images with sizes ranging between 100 × 100 and 1,000 × 1,000 pixels. These images were used to time several colordistance functions using the rbenchmark package (Kusnierczyk, 2012). Results are reported in Table 6.

**Table 6:** CIE Lab pairwise colordistance matrix for Figs. 3F–3J.

	F	G	H	I	J
F	–	–	–	–	–
G	72	–	–	–	–
H	224	155	–	–	–
I	**247**	181	39	–	–
J	232	174	73	48	–

**Note:**

Maximum score is in bold.

The most time-consuming function is convertColorSpace, which converts from RGB to CIE Lab space, since this is a non-linear transform (Hill, Roger & Vorhagen, 1997). The default behavior of colordistance is to use a random sample of 100,000 non-background pixels from a given image for CIE Lab conversion, since this typically takes fewer than 5 s and provides an accurate representation of the whole image.

### Examples

Unlike the artificial color images provided above, most real-world data involves comparing multiple colors across a range of both similarities and quantities. Quantitative, repeatable measurement and comparison of color profiles in images offers a valuable approach for answering a range of biological questions, which colordistance aims to make accessible with minimum requirements. Here, we present two analytical examples illustrating the different methods in colordistance, and how they can be used to quantitatively test color hypotheses about mimicry in butterflies and camouflage in flounder fish. The first example illustrates the utility of EMD as a distance metric in accounting for the similarity of non-identical colors using k-means clustering. The second example uses histograms and color range restriction.

The examples provided here use only one image per category (species, substrate, etc.) for simplicity and to keep the example datasets small, but a more robust analysis would use multiple images for each category, averaging color distributions together using the combineClusters function before computing a pairwise distance matrix. This approach will allow users to test color hypotheses with more statistically rigorous approaches.

Both examples use CIE Lab color space rather than RGB space, and use a D65 (indirect sunlight) standard illuminant to convert between RGB and CIE Lab space.

#### Example 1: Scoring mimicry in butterflies using earth mover’s distance and χ^2^ distance

**Figure 4:**
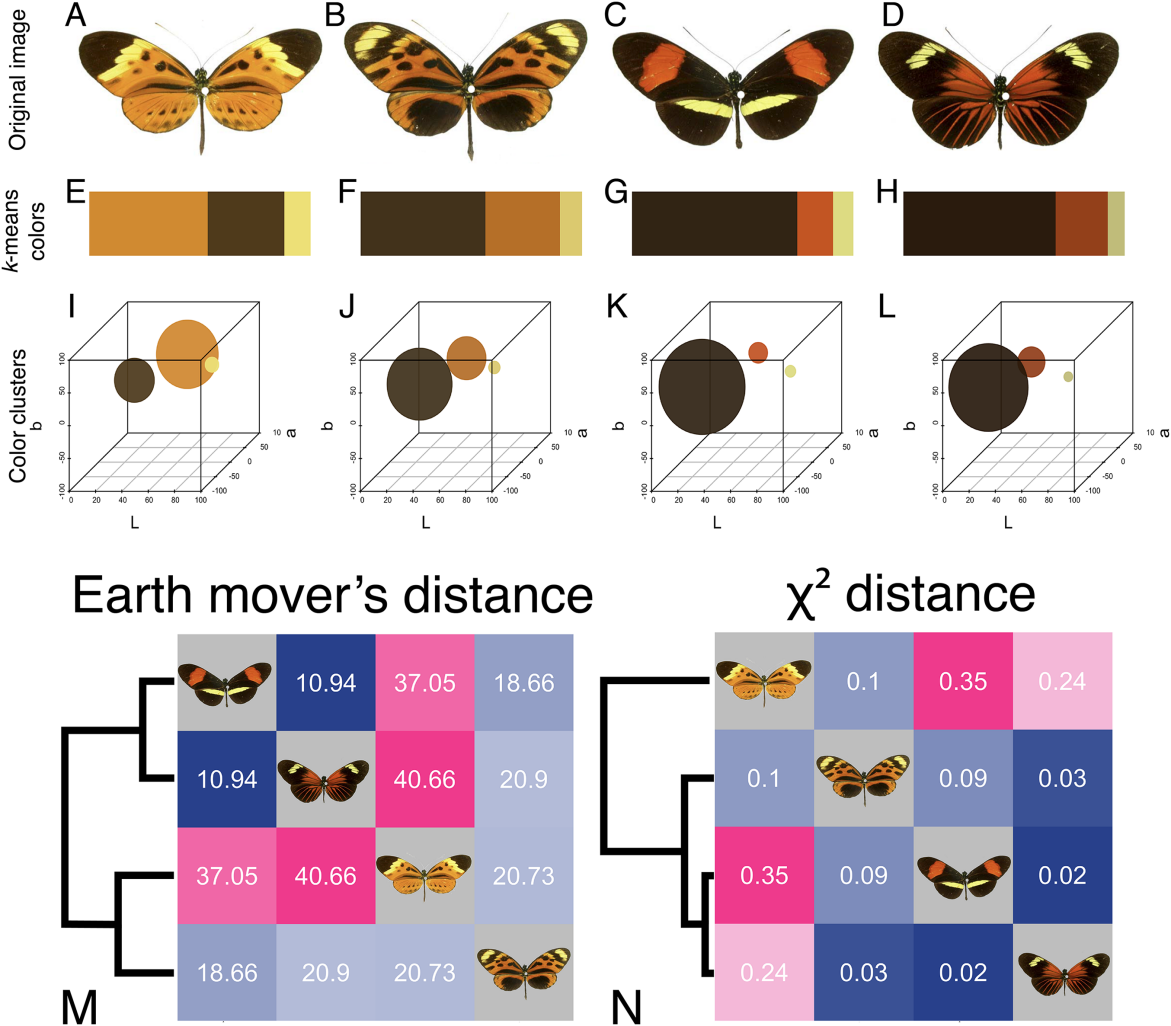
Color similarity analysis of *Heliconius* butterflies using earth mover’s distance and χ^2^ distance. (A–L) Butterfly images (A–D) with k-means clustering output as generated by getKmeansList, displayed as the default bar (E–H) and scaled to size in CIE Lab color space (I–L). (A) and (B) Two color morphs of *H. numata*; (C) and (D) two morphs of *H. melpomene*; (M) and (N) heatmaps of resulting color distance matrices, clustered by similarity, using earth mover’s distance (M) or χ^2^ distance (N). Dark blue is more similar, magenta is more dissimilar. Image credit for (A–D): Fig. 1 of Meyer (2006).

To illustrate how EMD outperforms more standard distribution comparison metrics, we used both EMD and χ^2^ distance to compare a set of four *Heliconius* butterflies with similar color palettes. *Heliconius* butterflies have been particularly well studied with respect to the evolution of color, pattern, and Müllerian mimicry (Kronforst & Papa, 2015; Enciso-Romero et al., 2017). Here, we illustrate the use of EMD with mimicry in two color forms of *Heliconius numata* and two color forms of *H. melpomene* (Figs. 4A–4D), as a way of testing the color similarity among forms in this system.

K-means clustering is useful for extracting the exact colors of an image when the number of colors is known in advance, rather than dividing a single patch of color into multiple bins (Ray & Turi, 1999). In this case, each butterfly appears to have three distinct colors (Figs. 4A–4D). To generate k-means fit objects for each image, the getKMeansList function is used, specifying 3 bins. The lower and upper arguments specify the lower and upper limits for RGB pixels to ignore as background—here, any pixels with R, G, and B values all between 0.8 and 1 (pale gray to pure white) will be ignored.

> kmeans_fits <- getKMeansList(“Butterfly_mimicry/”, bins = 3,    1
      lower = c(0.8, 0.8, 0.8), upper = c(1, 1, 1),        2
      color.space = “lab”, ref.white = “D65”,            3
      plotting = TRUE)                        4
> kmeans_list <- extractClusters(kmeans_fits, ordering = TRUE)    5

Line 1 returns a list of k-means fit objects using the kmeans function from the stats package and produces the bar plots shown in Figs. 4A–4D, with upper and lower bounds set to eliminate white pixels; these diagnostic plots are intended to help users determine whether the clustering accurately reflects the color distribution in the image. Line 2 extracts the clusters in the same format as getHistList for use with other colordistance functions; the ordering = TRUE flag uses an application of the Hungarian algorithm (Jonker & Volgenant, 1986) to order the most similar clusters in the same rows across dataframes. In this case, it ensures that all of the dark brown or black clusters are compared, the orange or red clusters are compared, and the yellow clusters are compared, rather than comparing the yellow cluster from one image to the black cluster from another. This is the default behavior of the function.

> emd_distance_matrix <- getColorDistanceMatrix(kmeans_list, method = “emd”)    1
> chisq_distance_matrix <- getColorDistanceMatrix(kmeans_list,         2
  method = “chisq”)                           3

Earth mover’s distance takes into account both the location and size of a given cluster when comparing one set of clusters to another, so that the final distance reflects the similarity of the clusters in both size and color (Rubner & Tomasi, 2013; Rubner, Tomasi & Guibas, 2000). χ^2^ distance, a more conventional metric for measuring the similarity of two distributions, compares bins only on the basis of size. To compare the two methods, the getColorDistanceMatrix function was used to compute a distance matrix for the clusters generated above using both EMD and χ^2^ distance. Lines 1 and 2 above produce the distance matrices in 4M-N. Note that the scales for each metric are different, and we will only be discussing the relative scores as indicated by the scaling of the colors in the heatmaps.

For either metric, butterflies C and D have the lowest distance (score as the most similar). Using χ^2^ distance, however, butterfly A is grouped outside of the rest of the photographs, because its orange cluster is considerably larger than the orange or red clusters of any of the other images, and its black cluster is much smaller (Fig. 4F). Using EMD, butterflies A and B score as more similar to each other than to either of the *H. melpomene* forms, because EMD takes into account the fact that the orange clusters for both images are closer in color space than they are to the red clusters of C and D. EMD balances color amount and color similarity when providing a distance score.

#### Example 2: Camouflage color matching in flounder using range-restricted histograms

**Figure 5:**
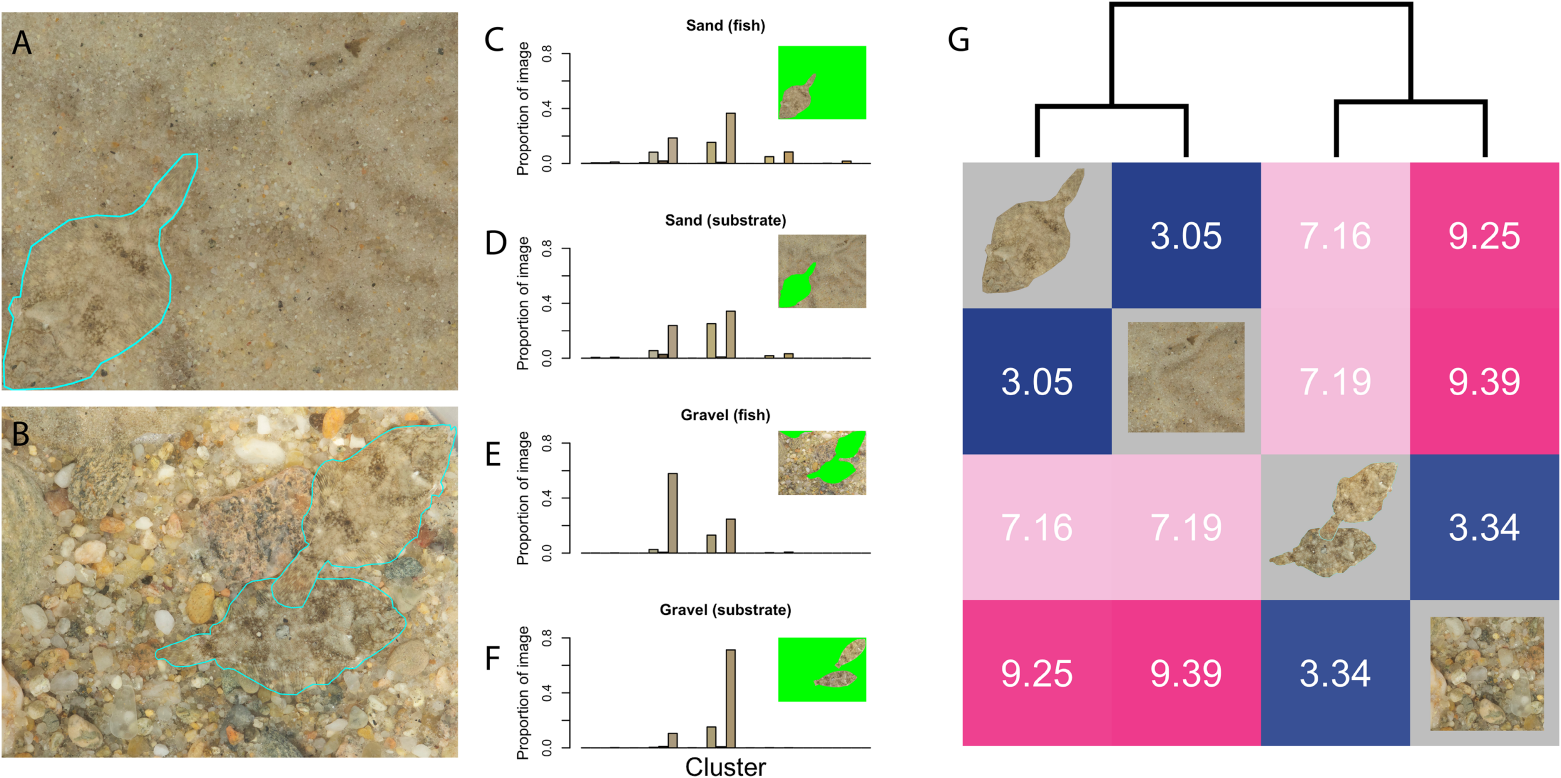
Background-matching analysis. (A) and (B) Flounder photographed on sand (A) and gravel (B), with fish outlined in cyan; (C–F) color histograms across a restricted color range in CIE Lab space, generated by getLabList, with insets indicating which part of the image was masked out in green and bars colored according to the average color of the pixels in each bin; (G) heatmap representation of distance matrix generated using getColorDistanceMatrix. Photo credit: H. Weller.

The ability of many organisms to display color patterns for camouflage against their surroundings provides insight into the relationships of organisms with their environments and with each other (Hanlon, 2007; Brady et al., 2015). Some species are capable of adaptive camouflage, in which the color pattern can be changed to match that of the environment or background. In this example, we illustrate the use of restricting the color binning range to test camouflage efficacy (fish matching the background) in winter flounder, *Pleuronectes americanus* (Fig. 5). Both sand and gravel substrates were analyzed, with an actively camouflaged flounder present on each background.

Because the colors are limited to tans and browns in both images, binning across all of a given color space will produce a large number of empty bins, and a small number of bins of extremely similar size and color across all four images, resulting in uninformative color distance calculations.

In order to produce a more informative histogram, the range of color space in which to divide pixels can be restricted. Here, inspection of pixel ranges in each color channel of CIE Lab space revealed that colors across all of the image had a-channel values between −20 and 40, and b-channel values between 0 and 50. Therefore, when calling the getLabHistList function to generate CIE Lab histograms for each image, these ranges were specified for the a.bounds and b.bounds arguments. Different numbers of bins for each channel—2 for luminance, 3 for a, and 5 for b—were also specified. Lower and upper ranges for ignoring bright green pixels are specified.

> flounder_hist <- getLabHistList(flounder, ref.white = “D65”,   1
       bins = c(2, 3, 5),                       2
       lower = c(0, 0.4, 0), upper = c(0.6, 1, 0.6),       3
       a.bounds = c(-20, 40), b.bounds = c(0, 50))        4
> flounder_distance_matrix <- getColorDistanceMatrix(flounder_hist) 5

The results of lines 1 and 2 are shown in Figs. 5C–5G. Camouflaged flounder score as most similar to the substrates on which they were photographed (Fig. 5G), quantitatively reflecting the species’ well-characterized ability to adjust color and pattern to a variety of backgrounds (Akkaynak et al., 2017). In each image, the fish were able to match the background color profile with the strikingly low distance of 3.05, while the sediments showed a difference of 9.39, more than three times as different. Study of the ability of organisms to change color either rapidly in an adaptive camouflage situation, or more gradually across life history stages may be a valuable application of this method. Because digital images are a poor proxy for visual systems that differ significantly from human visual sensitivities, however, caution should be used in interpreting the results.

In general, colordistance does not provide a categorical classification of images as similar or different, but instead a quantitative measurement of the degree of difference between each set of images. The final heatmap clusters images based on color similarity, but this clustering is intended as a visual tool for inspecting the results. Interpretation of the quantified differences will depend on the research question.

### Pipeline

The results in above examples can also be reproduced in their entirety using imageClusterPipeline, a function that produces a distance matrix from a set of images by calling on the binning, matrix calculation, and plotting functions in order, with specification options for every part of the pipeline.

For example 1:

> imageClusterPipeline(“Butterfly_mimicry/”,    1
          lower = c(0.8, 0.8, 0.8), upper = c(1, 1, 1),    2
          cluster.method = “kmeans”, kmeans.bins = 3,    3
          color.space = “lab”, ref.white = “D65”)    4

For example 2:

> imageClusterPipeline(“Flounder_camouflage/”,    1
          lower = c(0, 0.4, 0), upper = c(0.6, 1, 0.6),    2
          cluster.method = “hist”, hist.bins = c(2, 3, 5),    3
          a.bounds = c(-20, 40), b.bounds = c(0, 50),    4
          color.space = “lab”, ref.white = “D65”)    5

This function is convenient for quick tweaks or parameter checks, as the entire analysis can be run with a single line of code. The intermediate steps, however, may be more helpful for users performing other analyses.

### Comparison with the patternize R package

To illustrate the advantages of colordistance over other approaches for color and pattern analysis, we analyzed the same set of images of five parrotfish using both colordistance and patternize, an R package for quantifying color pattern variation (Van Belleghem et al., 2017). All images were captured under similar conditions against a gray background with a standard daylight flash. We used the default parameters of colordistance, including CIE Lab color space, histogram clustering with three bins per channel, and EMD for calculating color distances.

**Figure 6:**
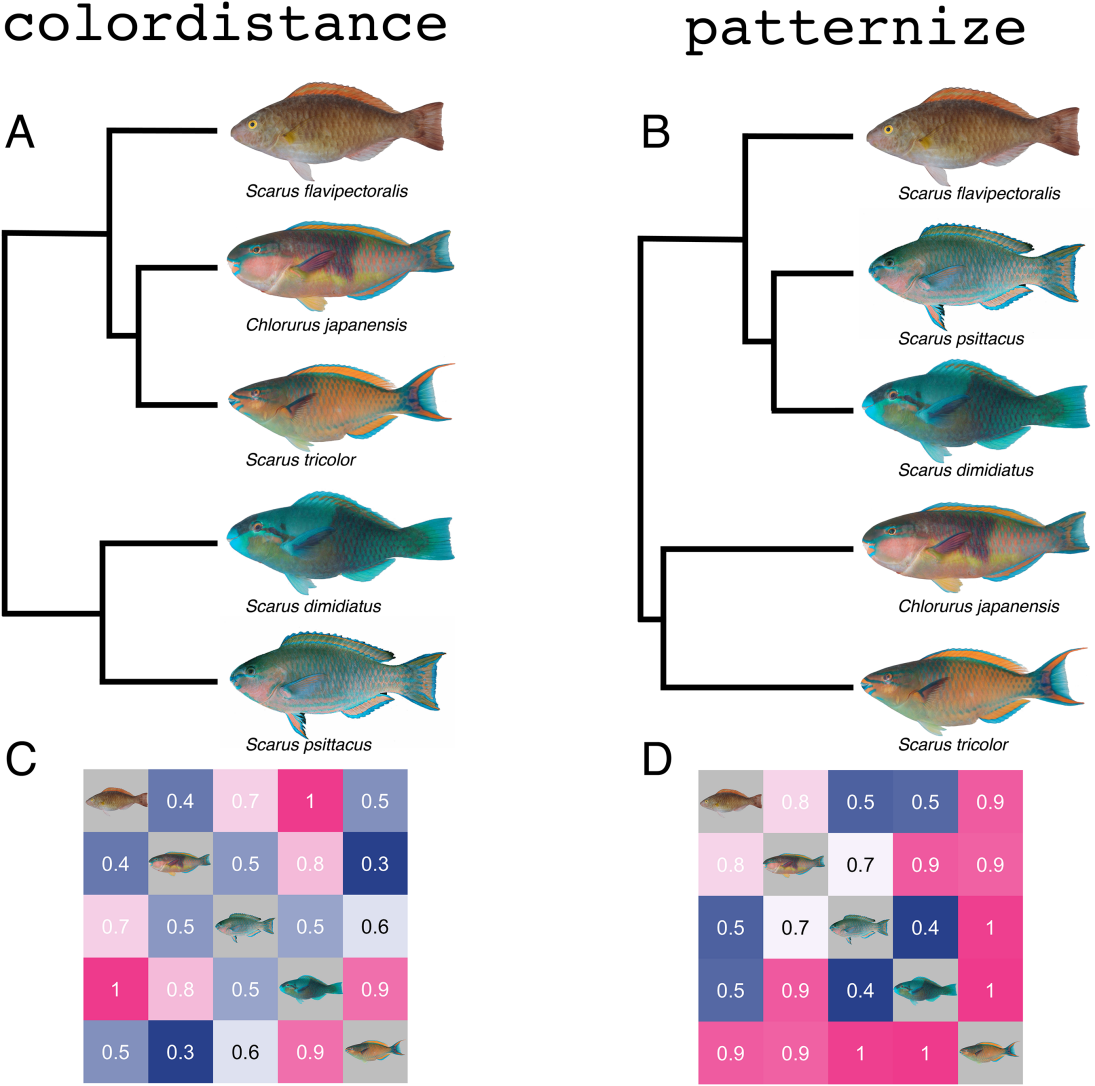
Similarity analyses produced by colordistance (A & C) and patternize (B & D) for the same set of parrotfish images. (A) and (B) Cluster analyses for color distance matrices produced by colordistance (A) and patternize (B). Branch lengths are proportional to the distances in the heatmaps below each dendrogram. Note the change in position of *Scarus flavipectoralis*. (C) and (D) Heatmap representations of normalized distance matrices produced by colordistance (C) and patternize (D), with species displayed in the same order for comparison between the heatmaps. Photo credit: M. Westneat/J. T. Williams.

For a similar comparison with patternize, we used homologous landmarks to align the images and predefined RGB colors based on earlier color sampling to compare the patterns of four dominant colors across species: green-blue, orange, pink, and brown (see methods). We chose to use predefined RGB colors because k-means clustering required the specification of so many clusters to account for the color variation in the more colorful images that it broke up dominant colors in other images, and the patterns in the images were not well-defined enough to use the watershedding approach. We then pooled the results of the principal component analyses for each color pattern into a single normalized distance matrix to compare similarity scores from the two packages (Fig. 6). The image of *Scarus flavipectoralis* is of an initial phase individual and therefore has no measurable green-blue coloration, which did not impact the colordistance analysis. Because patternize analyzes a single color at a time, however, the distance scores for *S. flavipectoralis* only include the results for the other three colors, which are present in the image (Fig. 6D).

The cluster analyses of both packages have relatively similar topologies: *S. dimidiatus* and *S. psittacus*, both of which are predominantly green-blue in color, group together, as do *Chlorurus japanensis* and *S. tricolor*, the two more colorful species. The location of *S. flavipectoralis* varies between the analyses: the colordistance analysis places it closer to *C. japanensis* and *S. tricolor*, while patternize places it closer to the predominantly green-blue species.

Other differences include that *S. flavipectoralis* and *S. dimidiatus* have the highest distance score in the colordistance analysis, while *S. tricolor* has the highest overall distance scores across all comparisons in patternize, with no distances below 90% of the maximum distance score. The lowest score in the colordistance analysis is between *C. japanensis* and *S. tricolor*, while in patternize it is between *S. dimidiatus* and *S. psittacus* (and *C. japanensis* and *S. tricolor* have a fairly high distance score despite grouping together in the cluster analysis). The numerical scores themselves are not reported, as the scales are not comparable—the patternize scores come from principal component analysis, while the colordistance scores come from EMDs in CIE Lab space.

In benchmark tests with the rbenchmark package (Kusnierczyk, 2012), colordistance performed the full analysis significantly faster than patternize (colordistance: 8.0 *±* 0.6 s; patternize: 158 *±* 8 s; *p* < 1.6 × 10^−6^ using a Student’s *t*-test), largely because colordistance discards spatial information, significantly speeding up computation. Implications of these results for the utility of colordistance compared to patternize are discussed below.

## Discussion

Colordistance provides an accessible tool for making quantitative color comparisons between images. The goal of the package is to provide a method for comparing both color quantity and similarity in an objective, repeatable way, without necessarily requiring homologous colors or even homologous morphologies. Given that many color pattern analyses do not have a method of scoring colors that are similar but not identical, colordistance provides a valuable additional analysis whenever there is considerable color variation across images in a dataset. Results provided by the package can be combined with other kinds of color and pattern toolkits to provide a comprehensive analysis of a system. The package is especially useful when considering systems where the colors across images are not necessarily homologous, and the degree of similarity between non-identical colors becomes more important. Here, we provide brief guidelines for choosing between the different color spaces, binning methods, and distance metrics in colordistance, and discuss how colordistance differs from similar packages and methods.

### Choosing parameters

The choices of color space, binning method, and distance metric used to analyze images in colordistance will all affect the final distance scores. Although default parameters generally perform well, and provide a reasonable trade-off between precision and efficiency, choosing appropriate parameters will depend on both the research question and the image set. Here, we provide brief guidelines for choosing parameters; more exhaustive discussions of color spaces, binning methods, and distance metrics are available in the literature (see Hill, Roger & Vorhagen, 1997; Ray & Turi, 1999; Rubner & Tomasi, 2013; Rubner, Tomasi & Guibas, 2000 and on the colordistance GitHub Pages site, https://hiweller.github.io/colordistance).

#### Color spaces

In general, users trade biological relevance for ease-of-use in choosing a color space. Of the three available color spaces (CIE Lab, RGB, and HSV) in colordistance, CIE Lab is generally the superior choice for measuring biologically relevant quantitative color similarities. Unlike RGB and HSV, CIE Lab is intended to be perceptually uniform, meaning that colors separated by an equal distance in CIE Lab space will be perceived as equally different. RGB and HSV color spaces are more computationally tractable because each channel in either color space ranges from 0 to 1; this allows for more consistent binning, even sampling, and universally scaled color distance measurements, since the absolute maximum distance will be fixed.

Although CIE Lab space is generally recommended for making quantitative color comparisons, it has several disadvantages compared to RGB or HSV. Because most digital images are stored in RGB format, working in CIE Lab space requires converting from RGB to CIE Lab. These conversions can be fairly time-consuming (Table 7), and require the specification of a white reference. It should also be noted that perceptually uniform color spaces like CIE Lab are designed to be uniform with respect to *human* color vision. The scaling in CIE Lab space therefore may not be perceptually uniform for other organisms, even those with trichromatic vision, because they may have significantly different peak visual sensitivities (Akkaynak et al., 2017; Hanlon, 2007). CIE Lab will still provide a closer approximation than a color space that doesn’t attempt perceptual uniformity, but caution should be used in interpreting the results. One possible workaround would be to use an image calibration, such as the software suite by Troscianko & Stevens (2015), to calibrate and normalize camera RAW files for non-human color visual systems before processing them with colordistance.

**Table 7:** Timing for the most time-consuming functions of colordistance.

	Function	Coefficient	*R*^2^	*p*-value
Loading images	loadImage	0.24 *s pixels*^−6^	0.97	<0.01
Converting from RGB to CIE Lab	convertColorSpace	57.3 *s pixels*^−6^	0.99	<0.01
Histogram binning	getLabHist	1.43 *s pixels*^−6^	0.97	<0.01
k-means binning	getKMeanColors	25.4 *s pixels*^−6^	0.97	<0.01

**Note:**

A total of five runs of each analysis were performed on an early 2015 MacBook Pro with a 2.7 GHz Intel Core i5 processor.

If the research question does not hinge on organismal color perception, however, RGB or HSV color spaces may be no more or less appropriate than a perceptually uniform color space. For example, if a user is attempting to quantify the proportion of discoloration on a series of leaves, any color space capable of separating the discolored and normal portions of the leaves in color space may be equally appropriate for quantifying the images. In this case, RGB or HSV would work well, and analyses in these color spaces will be considerably faster than in CIE Lab space. RGB is generally recommended over HSV because it is based on a tri-stimulus model of human color vision, with red, green, and blue channels that correspond approximately to human peak cone sensitivities (Byers, 2006).

#### Binning methods

Of the two binning methods, histogram binning and k-means clustering, histogram binning is the default because it makes fewer assumptions about color clustering in an image. Histogram binning counts how many pixels fall into each of a set of predetermined bounds, without the need for iteration, making it considerably faster than k-means clustering. Because the bins have the exact same bounds for each image, comparing bins across images is fairly straightforward, and empty bins account for the lack of specific colors in images. Histogram binning also has the advantage of retaining details, such as small accent colors in an image, rather than collapsing them into a larger cluster. However, it risks dividing up a single color into multiple bins, and can result in a large number of empty bins if the color range is not restricted (but see the flounder camouflage example above). Similarly, two different colors with pixels that happen to fall within the same bin will be averaged into a single color.

K-means clustering typically returns one cluster per dominant color in an image, provided an accurate number of clusters was specified (see Endler (2012) for methods of estimating the number of color classes). This can be useful when comparing a set of organisms or objects which have the same number of color classes, but different colors or amounts (see *Heliconius* example above). However, if users are attempting to compare objects with different numbers of colors, quantitative comparisons using k-means clusters requires either: (1) specifying a different number of clusters for each image and generating empty bins for the unmatched colors between images, or (2) specifying the highest required number of clusters for all images, typically breaking up colors across multiple clusters.

#### Distance metrics

Colordistance provides four metrics for quantifying the similarity of binned images, but EMD is recommended unless users have a specific reason for using one of the other three. Unlike the binning methods or color space, any of the given metrics will take approximately the same time to implement, since they require relatively little calculation unless a set of images is extremely large or uses hundreds or thousands of bins per image.

Of the four metrics, EMD is recommended for making general comparisons, as it takes both color similarity (relative location in color space) and amount (cluster size) into account to produce a single distance measurement for every pair of images in the dataset. EMD measures the difference between two images as a transport cost—how much work is required to reshape the distribution of image A so it resembles that of image B, or vice versa? Clusters of extremely different size require moving a large amount of data, and clusters in different parts of color space require moving data a long distance. Either one will increase the EMD, so that the highest EMD is the result of moving all of the data as far as possible across color space (e.g., an all-black cluster to an all-white cluster has to move 100% of the data the longest possible distance across RGB space).

Earth mover’s distance typically provides the best balance of color proportion and type in a set of images, and the resulting distance matrices reflect intuitive similarities in images (Rubner & Tomasi, 2013).

χ^2^ distance compares clusters only on the basis of size, even if two bins are slightly different colors (compare Figs. 4E and 4F). Color similarity is still taken into account in that using the same set of bins or setting ordering = TRUE for extractClusters() will guarantee that bins are comparable, but the relative color similarity of two bins is ignored beyond this. In practice, χ^2^ distance often performs about as well as EMD, except in cases where similar colors are placed into different bins, or have clusters of substantially different sizes. If users want to ignore these color differences, however—for example, when comparing images with the same expected color classes—χ^2^ distance is a viable choice.

The other two metrics, described in the “Color Distance Metrics” vignette in the package, calculate a distance score based on either (1) only color similarity, ignoring bin size, or (2) combining the size and color similarity scores according to specified weights. Although these metrics may be useful for certain questions or datasets, they don’t perform as well as either EMD or χ^2^ for general use, and are included only for specialized cases.

### Comparison with existing methods

Although color is notoriously subjective, it is also an indispensable tool for analyzing images. Computational solutions offer a repeatable, objective method for quantifying color with open-source tools, providing a statistically rigorous alternative to subjective analysis of images without requiring additional equipment beyond a personal computer. Colordistance is not intended as a superior replacement for more comprehensive image analysis tools, but as a complementary, easy-to-use option for including an analysis of color similarity that makes no assumptions about the homology of the images provided.

#### Comparison with patternize

Patternize uses either k-means, watershedding, or manual color specification approaches to segment images of organisms, and compares shapes of color patches to quantify the pattern similarities across a set of images. This method provides a rigorous comparison of pattern based on color, but has a number of limitations. Patternize works best when (1) the patterns share a relatively limited color palette across all objects, (2) the objects being compared have sharply defined color patterns, and (3) the objects have homologous shapes that can be aligned using a shape outline or a series of landmarks. It is therefore an extremely robust approach for comparing minor variations in color patterns within populations or between closely related species that share the same dominant colors (Kronforst & Papa, 2015).

In comparing parrotfish species, however, many of these conditions were fully or partially violated: some species are nearly uniform in color, while others display many distinct colors; many of the patterns are not sharply defined; and while species do share homologous landmarks, they vary considerably in their actual proportions, meaning that landmark alignment doesn’t ensure that the patterns themselves are actually aligned (Fig. 6). As a result, patternize alone cannot account for much of the interesting variation in this small example dataset. The initial phase *S. flavipectoralis*, for example, is almost entirely brown with an orange dorsal fin, yet it scores as relatively similar to the images of *S. psittacus* and *S. dimidiatus*, both of which are almost entirely green-blue. This is largely because they cluster together in the principal component analysis for the pink and orange patterns (*C. japanensis* and *S. tricolor* have substantial pink or orange on their flanks, while the other images do not), and because patternize does not account for the similarity of colors in different patterns.

In this case, even though the light brown coloration that dominates *S. flavipectoralis* is closer in color to the pinks and oranges of *C. japanensis* and *S. tricolor*, it groups as more similar to the green-blue fishes because all colors were treated as equally different. Similarly, *S. tricolor* scores as highly dissimilar from all of the other fishes, largely because much of its flank is classified as orange, while the other species have only orange accents; this ignores the similarity of the orange coloration. Moreover, many of the pattern elements themselves are not shared across the images—most of the fishes have a long stripe of some color on the dorsal fin, but only *S. dimidiatus* and *C. japanensis* have a dorsal saddle (in different colors), for example, which is not well-defined on either fish.

Colordistance, by contrast, takes into account the similarity and amount of each color in the images, while ignoring spatial information. While this fails to account for the shared pattern motifs, this method does account for the similarity of colors without the assumption that any set of colors is identical across images. Although the dominant brown coloration of *S. flavipectoralis* is not identical to the predominantly orange *S. tricolor* or the even more colorful *C. japanensis*, the similarity of their colors means that *S. flavipectoralis* scores as more similar to these two fishes than to either of the green-blue species, because orange and brown are much closer in CIE Lab space than brown and green-blue. For the same reasons, colordistance scores *S. tricolor* as quite similar to *C. japanensis* and *S. flavipectoralis*.

Neither of these analyses is necessarily superior to the other: they each reflect a different kind of information about the colors and patterns present in the images. The use of one over the other depends on the dataset and the research question. In this example, the colordistance analysis is more appropriate for questions about which species share similar color palettes despite wide variation in the actual pattern motifs displayed. An analysis with patternize would be more appropriate for answering questions about the similarity of the pattern motifs, perhaps ignoring color (in order to, e.g., compare dorsal saddles or facial markings of different colors). Interestingly, the similarity analyses produced by both methods are incongruent with parrotfish phylogeny (Smith et al., 2008), supporting that color on coral reef fishes is a highly adaptive trait (Hemingson et al., 2018). For broader comparative studies, such as comparing the diversity of shape and color across a large phylogeny or within an environment (Marshall et al., 2003), users may want to include an analysis of color similarity in addition to pattern similarity. Because both packages are available in R and can be used on the same datasets, users could combine information from both to weight both color and pattern similarity, for example, using colordistance to analyze the pattern similarity of patterns identified by patternize.

#### Color analyses with popular software

There are a number of computational tools that analyze color in digital images. The most popular tools for scientific analyses include the color plugins for Fiji/ImageJ (Schindelin et al., 2012), the MATLAB image processing toolkit (MATLAB Image Processing Toolbox, 2017), or the scikit-image and OpenCV libraries in Python (Van Der Walt et al., 2014; Bradski, 2000).

The image processing libraries available in MATLAB, Python, and C++ are geared largely toward explicit computer vision applications, rather than comparative pipelines. These libraries could be used to reconstruct any of the methods employed by colordistance by combining available clustering algorithms and appropriate distance metrics. However, the image analysis and statistical experience required to construct the pipeline from scratch may be prohibitive. Similarly, while ImageJ could be used to achieve the same results as colordistance, this would require images to be analyzed one at a time, and then for the histogram results to be analyzed in a separate program. This same result is achieved with a single line of code in colordistance, making it easier to test different color spaces, binning methods, and distance metrics, and to work with considerably larger image sets.

Colordistance is an R package, so it can easily be combined with other R packages and tools for color analysis or more general statistics. Because R is among the most popular coding languages in biological research, making these functions available in R allows users to make use of them without having to learn additional coding languages or to transfer the results of different analyses into new coding environments (R Core Team, 2018).

#### Other color comparison methods

Several other methods for comparing organismal color and pattern already exist, either as detailed protocols or software pipelines and packages.

A number of other R packages offer complementary functionality, including RImagePallette (extracts colors from images; Carlson (2016)), imager (a set of image processing tools; Barthelme (2017)), colorspace (mapping between color palettes; Zeileis, Hornik & Murrell (2009)), and pavo (spectral analysis; Maia et al. (2013)).

Endler (2012) provides a comprehensive analysis pipeline from image acquisition to color pattern geometry comparison. This method and similar ones are typically designed to answer specific questions about signaling, mate attraction, predator/prey camouflage, or pollination. A variety of tools could be used for different parts of the pipeline, including patternize and the other software tools mentioned above. Colordistance can be used as part of the analysis, specifically for determining and binning pixels into different categories, but the package isn’t designed to replicate or replace the spatial components of the analysis.

Instead, colordistance is intended to be one part of a larger color analysis toolbox, and can be used in conjunction with image segmentation or patch comparison methods to provide a more complete picture of how colors and patterns vary across images.

### Advantages and drawbacks

The major advantage of colordistance is that it has the same requirements as manual digital image classification (digital images and a computer), but provides a consistent, repeatable, objective alternative to subjective analysis, with a low barrier to entry. Because the analysis pipeline is reasonably fast and includes default parameters, an initial analysis is fairly quick and can be run in as little as a single line of code. This allows users to check for potential issues and tweak parameters to suit a dataset without spending hours or days re-running the analysis.

Users may employ multiple R packages for color processing, analysis and quantification of both color profile and pattern for a wide range of applications in biology. The colordistance package and tutorials, in combination with these other packages, provides an accessible method for researchers with a set of color images to perform a quantitative analysis of color similarity, all within the R environment.

However, colordistance is not a comprehensive analytical tool, and most notably does not perform any spatial analysis when considering the amount and similarity of colors in images. This means that two images with the exact same colors but completely different spatial distributions will receive the same similarity score as images with the same spatial distribution of colors. If pattern is a very important aspect of the analysis, then colordistance alone is not a sufficient tool, and should only be used as a complement to tools that are intended for spatial pattern analyses, such as patternize (Van Belleghem et al., 2017) or the method detailed by Endler (2012).

The package is also currently limited to a three-channel model, as all available color spaces in the package contain only three channels. This works well enough for most digital images, which are stored in a three-channel format, and for making comparisons through the lens of human vision, but it is not applicable for many animal models of color vision, since it is tailored for the visual sensitivities of human beings (Endler & Mielke, 2005, Akkaynak et al., 2017). Combining digital images with an ultraviolet sensor and using calibration tools to combine these channels (Troscianko & Stevens, 2015) is an excellent first step before attempting to use colordistance.

## Conclusion

Consistent, objective color comparisons are ideal for studying color in biology. Quantitative analyses are reproducible and scalable across datasets, without being prone to the subjective, variable, or inconsistent analyses that can result from more conventional categorizations of color. The examples presented here, illustrate how colordistance can produce quantitative answers to comparative questions about color in a flexible, user-friendly format. It is important to note that full color analyses would involve large samples of images (rather than the individual comparisons shown here) with appropriate statistical analyses of color profiles and distance metrics. The package provides a dynamic method of making quantified color comparisons between objects and computing distance matrices of color variation among objects. Color profile data and distance matrices are easily saved for incorporation into other R packages for statistics or phylogenetic comparative methods.

The method developed here is currently being used to analyze and compare the color palettes among families of coral reef fishes and other organisms, and should be applicable to analyses with a wide range of objectives. Although the package was developed for biological analysis, it can be used for any image set that requires quantitative color comparisons, from camouflage efficacy to trends in apparel. Current uses include an auto safety application, a study of soil color, a dominance study in lizards, and quantification of areas of fungal infection on plant leaves and bat wings.

Future development of the colordistance package will include expansion to include additional color channels (especially an ultraviolet channel), integration with landmark-based morphometric data sets, and user tools for partitioning objects into different regions. The analysis pipeline presented here could also be combined with pattern analysis software, such as the patternize R package (Van Belleghem et al., 2017), to compare both color and pattern similarities.

## Additional Information and Declarations

### Data Availability

The following information was supplied regarding data availability: GitHub (development version of code): https://github.com/hiweller/colordistance. GitHub (example images and code): https://github.com/hiweller/colordistance_examples. Comprehensive R Archive Network (CRAN) (stable release code only): https://CRAN.R-project.org/package=colordistance.
